# The sword of Damocles for the splenectomised: death by OPSI

**DOI:** 10.3205/000237

**Published:** 2016-08-29

**Authors:** Christian Georg Blumentrath, Nils Ewald, Jasmina Petridou, Uwe Werner, Barbara Hogan

**Affiliations:** 1 Department of Emergency Medicine, Muehlenkreiskliniken, General Hospital Luebbecke-Rahden, Germany; 2 Department of Internal Medicine, Muehlenkreiskliniken, General Hospital Luebbecke-Rahden, Germany; 3 Justus-Liebig-University Giessen, Germany; 4 Department of Medical Microbiology, Muehlenkreiskliniken, Minden, Germany; 5 Department of General and Abdominal Surgery, Muehlenkreiskliniken General Hospital Luebbecke-Rahden, Germany; 6 Group Departments of Emergency Medicine, Muehlenkreiskliniken, Minden, Germany

**Keywords:** overwhelming post-splenectomy infection (OPSI), septic shock, purpura fulminans, post-splenectomy vaccination, prevention of OPSI

## Abstract

The overwhelming post splenectomy infection (OPSI) in splenectomised patients is a rare but severe infection mostly caused by encapsulated bacteria.

We analyse the case of a 65-year-old female patient who was presented with clinical and laboratory findings indicating gastroenteritis. Two years years before admission, the patient underwent a splenectomy for a two stage splenic rupture following resuscitation for pulmonary embolism. Immunisation of the patient was complete and timely. As a result of the unspecific clinical presentation, there was a delay in administration of antibiotics. However, administration of antibiotics induced a fulminant shock. The patient died 4 hours after attending the hospital due to a pneumococcal sepsis.

The discussion highlights epidemiological and pathophysiological aspects and potential prevention strategies in the international context. Vaccination failed in our patient as the isolated pneumococcal strain (serogroup: 12F) is usually covered by the 23-valent pneumococcal polysaccharide vaccination (Pneumovax^®^). The case reported here indicates that there may be a potential benefit of prophylactic antibiotic treatment within the first 3 years after splenectomy for patients above the age of 65 years. Awareness of OPSI (prevention strategies, symptoms and treatment) among patients and their treating physicians is crucial for the improvement of prognosis. We partly address these issues in a standard operating procedure for the assessment of splenectomised patients in our departments of emergency medicine.

## Zusammenfassung

Die Post-Splenektomie-Sepsis ist eine seltene, häufig tödlich verlaufende Erkrankung bei splenektomierten Patienten, die überwiegend durch kapseltragende Bakterien verursacht wird. Wir berichten den Fall einer 65-jährigen Patientin, die sich aufgrund von seit 12 Stunden bestehendem, allgemeinen Krankheitsgefühl, Übelkeit, Erbrechen und Durchfall in der Notaufnahme vorstellte. Zwei Jahre zuvor war sie aufgrund einer fulminanten Lungenembolie reanimationspflichtig geworden. Im Anschluss daran entwickelte die Patientin eine zweizeitige Milzruptur. Die Milz wurde entfernt und empfohlene Impfungen zeitgerecht verabreicht. Die Zusammenschau der initial erhobenen Befunde machte die Diagnose einer Gastroenteritis wahrscheinlich. Dies wiederum führte zu einer verzögerten Antibiotikagabe. Die Antibiotikagabe wiederum löste ein fulminantes Schockgeschehen aus. Die Patientin verstarb 4 Stunden nach Krankenhausaufnahme an einer Post-Splenektomie-Pneumokokken-Sepsis. Die Diskussion widmet sich der Epidemiologie der Erkrankung, dem Verlauf der Erkrankung und den möglichen Präventionsstrategien. In der Zusammenschau kann die Aufklärung der betroffenen Patienten über die Notwendigkeit, auch bei geringen Infektionssymptomen ihren Arzt aufzusuchen nicht überbewertet werden. Auch die Bereitschaft der behandelnden Ärzte, auf den bloßen Verdacht hin bereits intravenös Antibiotika zu verabreichen, ist mitentscheidend. Wir haben daher eine Standard-Arbeitsanweisung für splenektomierte Patienten in unseren Notaufnahmen implementiert. Zudem wirft die Diskussion des Falles die Frage eines möglichen Nutzens einer prophylaktischen Antibiotika-Therapie bei über 65-jährigen Patienten auf (analog zu den Empfehlungen für Kinder).

## Introduction

Splenectomised patients face a threat which is frequently lethal. The overwhelming-post-splenectomy-infection (OPSI) has a lethality rate of ~50–70% [1]. The risk of OPSI correlates with age at and reason for splenectomy [1]. It is highest in children splenectomised for thalassemia major (8.2%) and drops to 1.1% in adults splenectomised for traumatic spleen rupture [1]. 

Usually, OPSI is caused by encapsulated bacteria: *Streptococcus pneumoniae*, *Haemophilus influenza* (HIB) and *Neisseria meningitides* [1]. However, *Escherichia coli*, *Pseudomonas aeruginosa*, *Capnocytophaga canimorsus* and other bacteria can also be causative [1]. 

Recommendations on vaccination of these patients, which includes pneumococcal, *Haemophilus influenza* type B, meningococcal group C conjugate and Influenza immunisation, partly anticipate this threat [1], [2]. Although there is no evidence of a potential benefit from life-long antibiotic prophylaxis for splenectomised adults, in the United Kingdom and other European countries this prevention strategy is generally recommended [1]. Di Sabatino et al. emphasize the need for a tailored antibiotic prophylaxis strategy among the inhomogeneous group of splenectomised adults [1].

The disease starts with unspecific symptoms [1]. Fever, nausea, vomiting and abdominal pain are characteristic [1]. The course of OPSI can mimic the Waterhouse-Friderichsen syndrome [1]. Treatment of choice consists of a combination of ceftriaxone and gentamycin [1], [2].

## Case report

Paramedics brought a 65 year old female patient to our department of emergency medicine as she was in a reduced general condition. The patient was severely obese (body mass index: 41 kg/m²), fully conscious (GCS: 15) and complained about nausea, vomiting, left-sided abdominal pain, severe weakness and a slight cough. The patient denied having dyspnoea and thoracic pain.

Two years prior to admission, pulmonary embolism resulted in cardiopulmonary resuscitation. After successful resuscitation, a splenectomy due to a traumatic two-stage splenic rupture was performed. The postoperative course in the intensive care unit was complicated by an angioneurotic edema and blood stream infection with *Serratia marcescens*, which was cured by ceftriaxone and piperacillin/tazobactam but resulted in prolonged weaning. The patient had fully recovered during the past 2 years after discharge from hospital.

Her further history included mild hypertension, recurrent upper gastrointestinal and urogenital bleeding following various anticoagulation treatments, which was based on esophagitis (stage IV, Savary and Miller), and diverticulosis of the descending and sigmoid colon. Consequently, she was receiving rivaroxaban 20 mg 1x1, torasemid 5 mg 1x1, ramipril 5 mg 1x1 and esomeprazole 40 mg 1x1. All vaccinations had been administered as recommended for splenectomised patients [2] including vaccination for *Streptococcus pneumoniae* (Pneumovax^®^).

On admission, she displayed tachypnea (25/min), tachycardia (116/min), a slightly impaired oxygen saturation (91%), normal blood pressure (112/76 mmHg) and fever (39.0 degree Celsius). 

Auscultation of the lungs was normal. Palpation of the abdomen enhanced the pain in the left part of the abdomen, suggestive for diverticulitis. No skin pathologies were observed. Apart from a sinus tachycardia, the electrocardiogram was normal. In particular, there was no link to myocardial infarction of pulmonary embolism.

The patient received intravenous paracetamol (1 g) and 1000 ml electrolyte-solution, which normalized the fever, tachypnea and tachycardia. We additionally administered 40 mg of pantoprazole.

Blood gas analysis revealed normal findings except for reduced oxygen saturation (81%), which lead to oxygen administration (4 l/min). Urine examination excluded a urinary tract infection. 

We performed a chest X-ray and computed tomography of the abdomen, which did not reveal any sign of a causative pathology. Laboratory analyses revealed no leucocytosis, normal lactate, elevated C-reactive protein (63 mg/l; normal: <5 mg/l), an elevated creatinine-level (1.5 mg/dl; normal <0.95 mg/dl) and marginalized positive troponin (123 µg/dl; normal <100 µg/dl). INR (1.27) was slightly impaired and PTT was normal. The patient was referred to our intensive care unit.

Two hours after admission to hospital, we started intravenous administration of 4 g ceftriaxone on suspicion of a highly feverish infection of unknown origin, accompanied by another 1,000 ml electrolyte-solution. 

Twenty minutes after administration of ceftriaxone, the patient’s condition deteriorated. Vomiting and diarrhoea preceded a generalized cyanosis. Oxygen saturation dropped, the patient became drowsy and short of breath while circulation still remained stable. 

We suspected a potential allergic reaction and administered subcutaneous adrenaline (0.5 mg), prednisolone (250 mg) and increased oxygen therapy, which primarily stabilized the patient. 

Re-evaluation for cardiac enzymes revealed no increase in troponin levels and assessment for pulmonary embolism showed only slightly elevated d-dimers (1.1 mg/dl; normal: <0.5 mg/l). 

Within 30 minutes, livid spots appeared on her skin which increased in size and number. The patient’s condition now required intubation, mechanical ventilation and additionally, continuous increase of intravenous noradrenalin application. Despite excellent oxygenation in blood gas analysis, cyanosis persisted. Four hours after admission to hospital, the patient died. 

Eight hours after collection, blood cultures became positive for *Streptococcus pneumoniae* (serogroup: 12F), and confirmed the differential diagnosis of OPSI retrospectively.

## Discussion

The lack of specific clinical symptoms and the fulminant course of the disease are frightening. Death by OPSI frequently occurs within the first 48 hours after admission [1], [2]. However, the fulminant course and outcome of the patient provided an enigma to the team of treating physicians and nurses. 

The clinical symptoms of the patient with absence of leucocytosis and moderately elevated C-reactive protein-levels indicated diagnosis of gastroenteritis. On admission, the patient did not match the criteria of severe sepsis [3]. 

Nevertheless, the initial treatment of the patient anticipated most of the recommendations of the international guidelines for the management of severe sepsis and septic shock: blood cultures before antibiotic therapy, imaging studies performed promptly to confirm a potential source of infection, administration of broad-spectrum antimicrobials therapy, initial fluid resuscitation with crystalloid (30 ml/kg) and referral to the intensive care unit [3]. We did not fully address the guidelines for the treatment of OPSI, which recommend ceftriaxone in combination with an initial single shot of gentamycin (5–7 mg/kg body weight) or, in case of a suspected abdominal focus: ciprofloxacin [1], [2]. Additional information: Gram coloration of buffy-coats from blood cultures may accelerate microbiological diagnosis [1], [2].

As seen in our patient, heavy vomiting and diarrhoea frequently indicate the onset of an endotoxin shock [4]. We presume that administration of antibiotic therapy released bacterial endotoxins, which resulted in a fulminant septic shock, characterized by cyanosis, hypotension and disseminated intravascular coagulation (DIC), as the isolated pneumococcal strain was fully susceptible to ceftriaxone [4]. Despite treatment of a potential allergic shock including the application of subcutaneous adrenaline and prednisolone, the patient’s condition deteriorated. 

In accordance with the guidelines for the treatment of severe sepsis and septic shock, we (a) intubated the patient which led to excellent oxygenation but despite (b) increase of fluid resuscitation and (c) application of intravenous norepinephrine in continuously increasing dosages the patient died in a septic shock [3], [5]. Administration of albumin and additional application of vasopressin or dobutamin might have been beneficial [3]. However, the septic shock was complicated by purpura fulminans, which is a frequent complication of pneumococcal sepsis in asplenic patients (19%) [3]. This complication is characterized by haemorrhagic infarction of the skin caused by disseminated intravascular coagulation and dermal vascular thrombosis, which initially appears as bluish or livid skin spots [5]. 

We seriously considered a cardiogenic and allergic shock as well as gastroenteritis aggravated by recurrent pulmonary embolism as potential differential diagnoses. The temporal link between application of ceftriaxone and the onset of shock-symptoms was causative for application of prednisolone, which is not recommended in the treatment of severe sepsis [3]. Retrospectively, an allergic shock is rather unlikely as the previous use of ceftriaxone in the patient had been well tolerated and the clinical and laboratory findings indicate septic shock.

Pulmonary embolism was probably not causative of the acute shock symptoms. Discrete elevation of d-dimers is being explained best by the infection [6]. In addition, the patient was protected by rivaroxaban. The slightly increased troponin-levels remained stable and were most likely due to the increased creatinine level [7]. Regrettably, echocardiography was not done. 

Relapse of infection due to *S. marcescens*, a rare cause of hospital-acquired infection which may cause outbreak-situations was unlikely in a sufficient degree [8]. 

Our patient was at a relatively low risk for OPSI [1]. In addition, her immunisation status was complete and compliant with guidelines [2]. Recommendations on vaccination of splenectomised patients includes immunisation for: Pneumococcal group 13 valent conjugate pneumococcal vaccination (Prevenar^®^) followed by 23-valent pneumococcal polysaccharide vaccination (Pneumovax^®^) 12 months later, repeated every 5 years), *Haemophilus influenza* type B (single dose), meningococcal group C conjugate (e. g. Nimenrix^®^ one initial dose repeated after 12 months, then every 5 years) and influenza (yearly) [2]. 

Serio et al. reported not a single case of OPSI in vaccinated patients in a cohort study which consisted mostly of patients which were at higher risk for OPSI (e.g. splenectomy for thalassemia or M. Hodgkin, and splenectomy in children) [9]. Admittedly, in contrast to the German guidelines, most of the patients in this study received antibiotic prophylaxis [2], [9]. According to German guidelines, prophylaxis with antibiotics (penicillin or erythromycin) should be given to children for the first three years after splenectomy and to adults until completion of immunisation [2]. Annual incidence of pneumococcal infection is high in children below the age of 1 year (8/100,000) and declines in children between the ages of 2–4 years (2.5/100,000) [10]. Incidence remains low in adults [10]. In the elderly, the annual incidence increases [10]. It is similar to children below the age of 1 year and the elderly between the age of 65 and 74 years (8/100,000) [10]. The annual incidence nearly doubles in elderly between the age of 75 and 84 years (14/100,000) [10]. Taking into account the increased risk of OPSI in the first 2 years after splenectomy and the annual incidence rates, a potential benefit of prophylactic antibiotic treatment within the first 3 years after splenectomy for patients above the age of 65 should be re-evaluated [2], [10]. 

Pneumovax^®^ was used for the vaccination of our patient 14 days after splenectomy. This vaccination covers several pneumococcal strains including the patient’s isolate (serogroup 12F). Conjugate pneumococcal vaccination (Prevenar^®^) followed by pneumococcal polysaccharide vaccination (Pneumovax^®^) 12 months later improves the response-rates to vaccination [1], [2], [11]. Response to immunisation 2 weeks after splenectomy is inferior to immunisation prior to splenectomy [1], [11]. Unfortunately, measurement of protective antibody levels was not done in our case.

Consequent immunisation of splenectomised patients, compliant to guidelines, is obligatory though not 100% protective [1], [2]. Immunisation may fail and additionally, the available vaccines do not cover all strains of the causative pneumococcal, meningococcal and haemophilus bacteria [1], [9]. Our patient died 16 hours after the onset of the first and unspecific clinical symptoms despite full vaccination. Awareness of the risk OPSI among patients and physicians remains low [5]. This is reflected by the generally poor follow-up immunisation rates of splenectomised patients [5]. As a consequence of the case we report here, we implemented standard operating procedures for the assessment of splenectomised patients in our departments of emergency medicine, which partly address these issues (see Attachment 1).

## Conclusion

Improved awareness of initial symptoms, the course of the disease, diagnosis and treatment of OPSI among splenectomised patients and physicians and immediate application of antibiotics in case of a suspected infection are the key to improving outcome prognosis. We adjusted our standard operating procedures for the assessment of splenectomised patients in our departments of emergency medicine and they now reflect this issue (see Attachment 1). The case reported here may indicate a potential benefit of prophylactic antibiotic treatment within the first 3 years after splenectomy for patients above the age of 65.

## Notes

### Competing interests

The authors declare that they have no competing interests.

### Author’s contributions

CGB conceived the idea for this case report, performed literature search and drafted a first version of the manuscript. All authors reviewed the results of the literature search and contributed equally to the final version of the manuscript. All authors read and approved the final manuscript.

### Ethical statement

The husband of the patient gave written informed consent for the publication of the case report in agreement with the other family members.

### Acknowledgement

The authors thank the team of doctors and nurses of the departments of emergency medicine and the intensive care unit, Muehlenkreiskliniken, General Hospital Luebbecke-Rahden, Rahden Site. We acknowledge the determination of the pneumococcal strain by the national reference centre for streptococci, Institute of Medical Microbiology, University Hospital Aachen, Pauwelstraße 30, Aachen, Germany. We obtained written informed consent for the publication of the case details from the patient’s husband.

## Supplementary Material

Standard operating procedures for the assessment of splenectomised patients in departments of emergency medicine
