# Supplementary material for: The sword of Damocles for the splenectomised: death by OPSI
Source: Ger Med Sci. 2016 Aug 29;14:Doc10. doi: 10.3205/000237 (PMC5004600; doi:10.3205/000237)
Supplement: Standard operating procedures for the assessment of splenectomised patients in departments of emergency medicine [file GMS-14-10-s-001.pdf]

Standard Operating Procedure:
Assessment of splenectomised patients in departments of emergency medicine

Background

Overwhelming post-splenectomy infection (OPSI) is a life-threatening event. Therefore, splenectomised patients deserve special attention in departments of emergency medicine. In order to reduce the risk of OPSI, all recommended vaccinations (HIB, meningococcal, pneumococcal and influenza vaccination) need to be renewed regularly. The typical symptoms of OPSI are unspecific: flu like symptoms, fever, abdominal complaints, nausea, vomiting and diarrhoea. Note: elderly patients may not display typical symptoms of infection. These patients may only display an altered mental status.

First view

Immediate triage and information sent to the attending physician

↓

Does the triage (blood pressure, heart rate, temperature, breathing rate, or pain), history or physical examination of the patient reveal any symptoms indicating an infection?

Approach

yes → Proceed to page 2: Assessment of splenectomised patients with suspicion of OPSI

No → Proceed to page 3: Assessment of splenectomised patients with no suspicion of OPSI

Literature/Source

The recommendations of this SOP derive from: Engelhardt M et al. Prävention von Infektionen und Thrombosen nach Splenektomie oder funktioneller Asplenie. DGHO Onkopedia. Accessible online: https://www.onkopedia.com/de/onkopedia/guidelines/praevention-von-infektionen-und-thrombosen-nach-splenektomie-oder-funktioneller-asplenie/@@view/html/index.html

Standard Operating Procedure:
Assessment of splenectomised patients with suspicion of OPSI in departments of emergency medicine

Immediate Diagnostics and Therapy

OPSI is a life-threatening emergency. The lethality rate is high (50-70%). Most patients die within the first 24-48 hours after admission to hospital.

↓

Secure vital functions and surveillance of the patient. Implement a large intravenous line. Initiate fluid therapy. ECG. Initiate blood and urine analysis (blood count, blood cultures, blood gas analysis including lactate, creatinine kinase, troponin, d-dimers, ALAT, ASAT, bilirubin, creatinine, electrolytes, procalcitonin, c-reactive protein, coagulation analysis, urine analysis and culture)

↓

Extended Diagnostics

Are there any findings (history of the patient, thorough physical examination, immediate diagnostics) indicating any other severe disease or life-threatening cause of the displayed symptoms?

Yes → Include your suspicion into your approach towards the patient

No → Perform chest X-ray, ultra sound examination of the abdomen, echocardiography. computed tomography?

(Both boxes are linked by a two-way arrow; both lead to the next step.)

↓

Specific Therapy

Initiate intravenous administration of antibiotics. Suspected abdominal focus: ceftriaxone and ciprofloxacine.
Otherwise ceftriaxone and single shot gentamycine (5-7mg per kg body weight).

Beware of potential contraindications and recommendations concerning the need for adjusting dosages.

Referral of the patient to the intermediate or intensive care unit

Standard Operating Procedure:
Assessment of splenectomised patients with no suspicion of OPSI in departments of emergency medicine

Information

Is the patient sufficiently informed about the potential risks (OPSI and thrombosis) which derive from splenectomy?

No

Give sufficient information about the risk of OPSI and thrombosis and resulting consequences according to the recommendations given by Engelhardt M et al. Prävention von Infektionen und Thrombosen nach Splenektomie oder funktioneller Asplenie. DGHO Onkopedia.

Yes

State of vaccination

Did the patient receive all necessary vaccinations according to the recommendations given by Engelhardt et al. (Literature page 1)?

No

Give advice concerning necessary vaccinations to the patient and to the next treating physician according to the recommendations given by Engelhardt et al. (Literature page 1)

Yes

Approach

Further assessment of the patient depends on the corresponding standard operating procedure addressing your suspicion
